# STIM1 plays an important role in TGF-β-induced suppression of breast cancer cell proliferation

**DOI:** 10.18632/oncotarget.7619

**Published:** 2016-02-23

**Authors:** Huanyi Cheng, Shiqiang Wang, Renqing Feng

**Affiliations:** ^1^ State Key Laboratory of Membrane Biology, Department of Biochemistry and Molecular Biology, College of Life Sciences, Peking University, Beijing 100871, China

**Keywords:** TGF-β, SOCE, STIM1, cell proliferation, breast cancer

## Abstract

Store-operated calcium entry (SOCE) signaling is involved in cancer progression. Stromal interaction molecule 1 (STIM1) triggers store-operated calcium channels to induce SOCE. Transforming growth factor-β (TGF-β) influences a wide range of cellular behaviors, including cell proliferation. However, little is known about the relationship between calcium signaling and TGF-β signaling in cancer cell proliferation. Here, we found that TGF-β induced cell cycle arrest at the G0/G1 phase and suppressed cell proliferation in MDA-MB-231 and MCF-7 breast cancer cells. These effects were impaired by extracellular Ca^2+^ chelator EGTA or SOCE specific inhibitor SKF96365 in MDA-MB-231 cells. Treating MDA-MB-231 cells with TGF-β for 24 and 48 h markedly decreased STIM1 expression and thapsigargin-induced SOCE. A transcriptional inhibitor of STIM1, Wilm's tumor suppressor 1 (WT1), was upregulated in TGF-β-treated MDA-MB-231 cells, and knockdown of WT1 expression partially restored the TGF-β-induced downregulation of STIM1. Stably overexpressing STIM1 in MDA-MB-231 cells restored the TGF-β-induced effects. The p21 mRNA level increased in SKF96365- or TGF-β-treated MDA-MB-231 cells, whereas that for cyclin E1 decreased. Our findings demonstrate for the first time that STIM1 and SOCE are involved in the TGF-β-induced suppression of cell proliferation. Furthermore, our studies also provide a new approach to inhibit breast cancer cell proliferation with small molecules targeting STIM1 and SOCE.

## INTRODUCTION

Calcium, a ubiquitous second messenger, plays an important role in the development and maintenance of many physiological functions [1, 2]. The intracellular calcium concentration is precisely regulated, and aberrant Ca^2+^ regulation has been linked to various diseases [3, 4]. In many cell types, calcium influx is essential for regulating a variety of distinct processes involving enzymatic activity, gene expression, apoptosis, and cell proliferation [3, 5]. In nonexcitable cells, including most cancer cells, store-operated calcium entry (SOCE) is the predominant Ca^2+^ entry pathway [6]. Previous studies have reported that Orai and portions of the canonical transient receptor potential cation (TRPC) channel are involved in the store-operated calcium influx complex [2, 7]. Stromal interaction molecule (STIM) serves as a calcium sensor that triggers the Ca^2+^ influx responsible for replenishing the depleted state of the endoplasmic reticulum Ca^2+^ stores. For example, STIM1 is required for the attenuation of PMCA-mediated Ca^2+^ clearance during T-cell activation [8–10]. Recently, several studies have demonstrated that Orai1 and STIM1 are involved in cancer progression [6]. The levels of Orai1 and STIM1 are increased in therapy-resistant ovarian carcinoma cells [11], and they mediate breast tumor cell migration and metastasis [12]. STIM1 also regulates cell growth, migration, and angiogenesis in cervical cancer cells [13], and STIM1 knockdown suppresses cell proliferation and tumorigenicity in human epidermoid carcinoma A431 cells [14]. Furthermore, the overexpression of STIM1 promotes cell migration and upregulates cyclooxygenase-2 expression in colorectal cancer cells [15]. However, the molecular mechanism for the STIM1 regulation of cancer progression remains unclear, especially in the proliferation of breast cancer cells.

Transforming growth factor-β (TGF-β), a pleiotropic cytokine, influences a wide range of cellular behaviors, including cell proliferation, differentiation, and apoptosis [16, 17]. In the tumor microenvironment, TGF-β affects cell proliferation and cancer progression [18]. TGF-β signaling has a dual role in cancer progression. In the early stages of tumorigenesis, TGF-β acts as a tumor suppressor to inhibit the growth of cancer cells; at advanced stages, TGF-β induces the epithelial-mesenchymal transition and promotes cell migration, invasion, and metastasis [16, 19, 20]. Calcium signaling serves as a bridge to link signals from the tumor microenvironment with specific cellular responses, which involves a network of signaling pathways [21, 22]. For example, epidermal growth factor (EGF)-stimulated cervical cancer cell migration requires STIM1 expression, and the inhibition of SOCE suppresses EGF-induced migration and eliminates extravasation from the vasculature in nasopharyngeal carcinoma cells [13, 23]. In MDA-MB-468 breast cancer cells, SOCE is involved in EGF-induced epithelial-mesenchymal transition [24], and EGF-mediated cell growth requires Orai1 and STIM1 in ARPE-19 cells [25]. However, the relationship between cytokine-mediated cell behaviors and calcium signaling is still unclear, especially in the TGF-β-induced suppression of cell proliferation and SOCE signaling. To the best of our knowledge, there is no report on the involvement of SOCE in the TGF-β-induced suppression of breast cancer cell proliferation.

In the present study, we investigated the roles of SOCE and STIM1 in TGF-β-induced cell cycle arrest at the G0/G1 phase and suppression of proliferation in MDA-MB-231 cells. We found that the extracellular Ca^2+^ chelator EGTA or the SOCE specific inhibitor SKF96365 impaired TGF-β-induced cell cycle arrest and suppression of cell proliferation in MDA-MB-231 cells. Our results demonstrated that TGF-β treatment decreased SOCE by downregulating STIM1 expression in MDA-MB-231 cells. In addition, our findings showed that the stable ectopic overexpression of STIM1 in MDA-MB-231 cells restored the TGF-β-induced cell cycle arrest at the G0/G1 phase and suppressed cell proliferation to nearly control values. We also studied the molecular mechanism mediating the STIM1 involvement in TGF-β-induced arrest of the cell cycle and suppression of cell proliferation. Taken together, our findings demonstrate for the first time that TGF-β suppresses STIM1 expression by upregulating WT1 expression. Our results also suggest that STIM1 plays an important role in TGF-β-induced suppression of cell proliferation and that specific small molecules targeting SOCE may be potential candidates for cancer therapy.

## RESULTS

### EGTA impairs TGF-β-induced suppression of cell proliferation

To determine the effect of TGF-β on cell proliferation in human breast cancer cells, the viability of MDA-MB-231 and MCF-7 cells was evaluated with an MTT assay after exposure to TGF-β for 3 and 4 days. The results revealed that TGF-β inhibited the growth of MDA-MB-231 and MCF-7 cells (Supplementary Figure S1A and B). We also examined TGF-β-induced cell cycle arrest. After TGF-β treatment for 3 days, MDA-MB-231 and MCF-7 cells arrested at the G0/G1 phase of the cell cycle, with a concomitant loss of the S- and G2/M-phase cell populations (Supplementary Figure S1C-F).

**Figure 1:**
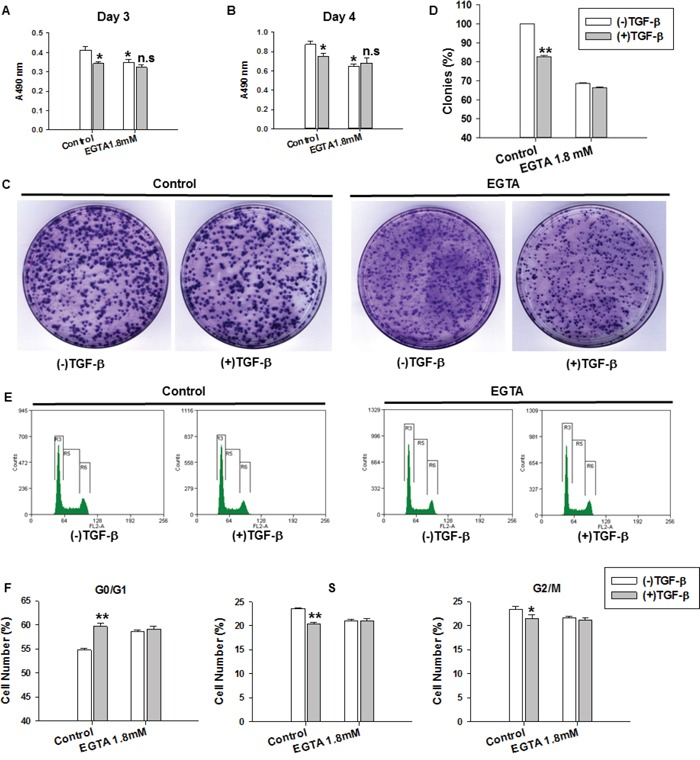
TGF-β-induced suppression of cell proliferation, cell colony formation and cell cycle arrest were impaired with EGTA treatment **A-B.** MDA-MB-231 cell proliferation was determined by MTT assay after various treatments for 3 and 4 days. Cell proliferation was expressed as the absorbance values. *P<0.05 VS. Control group without TGF-β treatment, n.s (not significant) VS. EGTA 1.8mM treatment group without TGF-β treatment. **C.** Colony formation assay in MDA-MB-231 cell with various treatments. **D.** The number of colonies was measured by the software Lane 1D. Numbers of colonies was quantified in 3 dishes from each treatment group. Results are the means ± SE from 3 independent experiments plotted as percent (%) colonies relative to control group. **E.** Cell cycle distribution was detected by flow cytometry in MDA-MB-231 cells with various treatments for 3 days. **F.** The statistical analysis of cell cycle distribution in G0/G1, S and G2/M phases. TGF-β and EGTA induced G0/G1 arrest. TGF-β-induced cell cycle arrest was impaired by EGTA (1.8 mM). Data represent the means ± SE of three independent experiments. *P < 0.05, **P < 0.01 compared with control group.

To understand the role of calcium signaling in cell proliferation, we treated MDA-MB-231 cells with different concentrations of the extracellular calcium chelators EGTA and BAPTA and then cultured the cells for the indicated time. Cell viability was measured with an MTT assay. As shown in Supplementary Figure S2A-D, EGTA and BAPTA (1.8 and 2.0 mM) significantly decreased cell proliferation on day 3 and 4. To determine whether calcium signaling was involved in the TGF-β-induced suppression of cell proliferation, we treated MDA-MB-231 cells with TGF-β (5 ng/ml), EGTA (1.8 mM), or TGF-β (5 ng/ml) plus EGTA (1.8 mM). Cell viability was measured with an MTT assay after treatment for the indicated time. TGF-β treatment significantly decreased cell proliferation (Figure 1A and 1B) compared with the control. After inhibiting Ca^2+^ influx, however, EGTA impaired the TGF-β-induced suppression of cell proliferation (Figure 1A and 1B).

Cell proliferative activity was then assessed using a colony formation assay. The colony formation capacity of MDA-MB-231 cells was estimated on day 12 using different treatment conditions. As shown in Figure 1C and 1D, the colony formation efficiency of MDA-MB-231 cells in the TGF-β-treatment group was significantly reduced compared with those in the control group. This effect was nearly abolished by inhibiting Ca^2+^ influx with EGTA (1.8 mM).

To further elucidate the growth suppressing effect of TGF-β on MDA-MB-231 cells, we performed cell cycle distribution analysis using flow cytometry after TGF-β treatment for 3 days. The cell cycle analysis results demonstrated that TGF-β (5 ng/ml) and EGTA (1.8 mM) induced G0/G1 arrest in MDA-MB-231 cells. The TGF-β-induced G0/G1 arrest was nearly abolished by EGTA (Figure 1E and 1F). These results indicate that Ca^2+^ influx is involved in the TGF-β-induced suppression of cell proliferation.

### TGF-β treatment decreases store-operated Ca^2+^ influx in MDA-MB-231 cells

**Figure 2:**
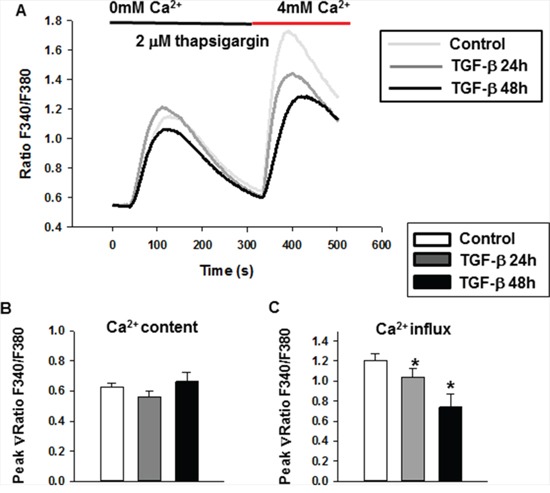
The store-operated Ca2+ entry decreased after TGF-β treatment in MDA-MB-231 cells **A.** The cytosolic Ca^2+^ concentration was measured in Fura 2-AM-loaded MDA-MB-231 cells. The dynamic intracellular Ca^2+^ concentration was shown as the ratio of F340/F380. **B.** The quantitative analysis of Ca^2+^ content induced by TG. **C.** The quantitative analysis of Ca^2+^ influx. For this assay, three individual experiments (about 10 cells each time, totally more than 30 cells for each group) were performed. Data were expressed as means ± SE. Significance was assessed using one-way ANOVA with Bonferroni's multiple comparisons post-tests, *P < 0.05.

To determine the role of calcium signaling in the TGF-β-induced suppression of cell proliferation, we measured SOCE, a major calcium entry pathway in nonexcitable cells [26]. Fluorescent dye Fura 2-AM was used to monitor the dynamic intracellular Ca^2+^ concentration. Because store-operated Ca^2+^ channels are activated when the endoplasmic reticulum Ca^2+^ store is depleted, thapsigargin (TG) was used to deplete the endoplasmic reticulum Ca^2+^ store in the absence of extracellular Ca^2+^ (0 mM Ca^2+^). The Ca^2+^ influx was then measured after the addition of extracellular Ca^2+^ (4 mM). The Ca^2+^ content and SOCE amplitude (Ca^2+^ influx) were evaluated at the peak of the F340/F380 ratio values after subtracting the basal ratio value. The experimental protocol is presented in Figure 2A. As shown in Figure 2B, treatment of MDA-MB-231 cells with TGF-β for 24 and 48 h did not affect the TG-induced Ca^2+^ release from the endoplasmic reticulum (Ca^2+^ content) under Ca^2+^-free conditions (Figure 2B). However, TGF-β significantly decreased the Ca^2+^ influx through SOCE signaling in the presence of extracellular Ca^2+^ (Figure 2C). Based on these results, we conclude that a reduction in functional SOCE might be involved in the TGF-β-induced suppression of cell proliferation.

### Blocking SOCE impairs TGF-β-induced cell cycle arrest and the suppression of cell proliferation

**Figure 3:**
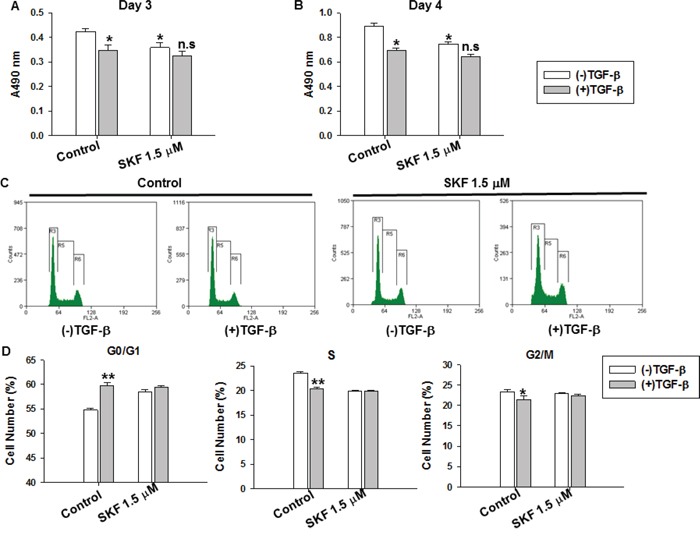
The specific SOCE inhibitor SKF96365 impaired TGF-β-induced suppression of cell proliferation and cell cycle arrest in MDA-MB-231 cells **A-B.** Cells were cultured with DMEM supplemented with 10% FBS containing TGF-β (5 ng/ml), SKF96365 (1.5 μM), TGF-β (5 ng/ml) plus SKF96365 (1.5 μM) respectively for 3 and 4 days. The cell proliferation was measured by MTT assay. *P<0.05 VS. Control group without TGF-β treatment. n.s (not significant) VS. SKF 1.5 μM treatment group without TGF-β treatment. **C.** The cell cycle distribution after 3 days' culture was detected by flow cytometry, and the percentage of the cells at G0/G1, S and G2/M phase with various treatments was analyzed. **D.** The statistical analysis of cell cycle distribution at G0/G1, S and G2/M phases. For this assay, bar graphs showed means ± SE for at least three independent experiments. Significance was assessed using student's t-test, *P < 0.05.

SOCE signaling has been implicated in cell proliferation [5]. We used the SOCE inhibitor SKF96365 to determine whether SOCE was involved in the TGF-β-induced suppression of cell proliferation. The proliferation of MDA-MB-231 cells treated with different concentrations of SKF96365 was analyzed with an MTT assay (Supplementary Figure S3A and B). MDA-MB-231 cells were treated with TGF-β (5 ng/ml), SKF96365 (1.5 μM), or TGF-β (5 ng/ml) plus SKF96365 (1.5 μM), and cell viability was measured with an MTT assay after culturing for 3 and 4 days. Treatment with SKF96365 nearly abolished the TGF-β-induced suppression of cell proliferation (Figure 3A and 3B). We also tested additional concentrations of SKF96365 with or without TGF-β (Supplementary Figure S3C and D). The results showed that treatment of MDA-MB-231 cells with SKF96365 and TGF-β did not have a synergistic effect.

We performed cell cycle distribution analysis using flow cytometry 3 days after different treatment conditions. As shown in Figure 3C and 3D, TGF-β (5 ng/ml) and SKF96365 (1.5 μM) induced cell cycle arrest at the G0/G1 phase in MDA-MB-231 cells. The percentage of cells arrested at the G0/G1 phase was higher in the TGF-β- (**P < 0.01) and SKF96365- (**P < 0.01) treated groups than in cells of the control group. Treatment with SKF96365 (1.5 μM) nearly abolished the TGF-β-induced cell cycle arrest (Figure 3C and 3D). These results indicate that SOCE is involved in TGF-β-induced cell cycle arrest and the suppression of cell proliferation.

### TGF-β regulates SOCE-related gene expression

**Figure 4:**
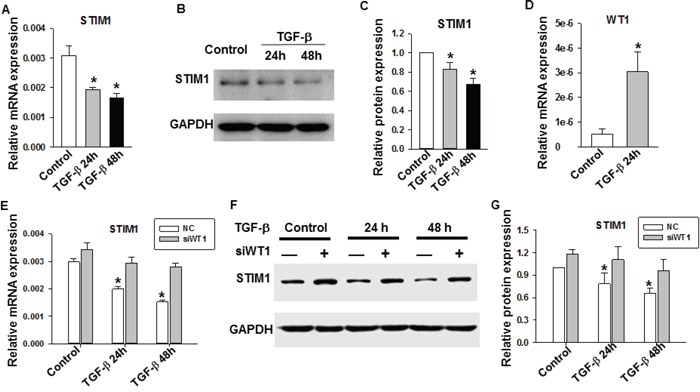
The role of TGF-β on *STIM1* and *WT1* gene expression in MDA-MB-231 cells **A.** Assessment of *STIM1* mRNA levels by qRT-PCR after TGF-β treatment for 24 and 48 h. **B.** Assessment of STIM1 protein expression level by Western blot analysis. **C.** The statistical analysis of STIM1 protein expression level referenced to GAPDH. **D.** Assessment of *WT1* mRNA levels by qRT-PCR after TGF-β treatment for 24 h. **E.** Assessment of *STIM1* mRNA levels by qRT-PCR after WT1 siRNA knockdown and TGF-β treatment. *P<0.05 VS. Control and NC group. **F.** Assessment of STIM1 protein expression level by Western blot analysis after WT1 siRNA knockdown and TGF-β treatment. **G.** The statistical analysis of STIM1 protein expression level referenced to GAPDH in different groups. *P<0.05 VS. Control and NC group. Data were representative of three independent experiments and bar graphs represented as the means ± SE. Significance was assessed using one-way ANOVA with Bonferroni's multiple comparisons post-tests, *p < 0.05.

To explore the molecular mechanisms mediating the TGF-β-induced reduction in SOCE amplitude, we performed qRT-PCR to quantify the mRNA expression levels of SOCE-related genes, including calcium release-activated calcium channels (*Orai1*, *Orai2*, and *Orai3*), stromal interaction molecules (*STIM1* and *STIM2*), and transient receptor potential cation (*TRPC1-7*) channels [22, 27]. For this assay, MDA-MB-231 cells were pretreated with TGF-β (5 ng/ml) for 24 and 48 h, and the relative mRNA expression levels were determined by qRT-PCR. As shown in Figure 4A, a significant decrease in the *STIM1* mRNA expression level was detected after treatment with TGF-β for 24 and 48 h. The mRNA expression levels of *Orai3* and *TRPC3* decreased only after TGF-β treatment for 48 h, whereas the levels of the other genes examined were not significantly different compared to control levels (Supplementary Figure S4A-H).

To assess which genes were most important in SOCE signaling in MDA-MB-231 cells, absolute quantitation of SOCE-related genes was conducted using RT-PCR. Our results indicated that *Orai1* and *TRPC1* were the primary SOCE-related channel subtypes in MDA-MB-231 cells (Supplementary Figure S5A-C). It was previously reported that the *Orai3* channel selectively mediates SOCE signaling in estrogen receptor α-positive breast cancer cells [28]. Therefore, we focused on determining whether there was a change in the STIM1 protein level after TGF-β treatment. Western blot analysis results revealed a significant decrease in the STIM1 protein level in TGF-β-treated cells compared with that in untreated cells (Figure 4B and 4C). These results suggest that STIM1 might be involved in the TGF-β signaling pathway.

### Mechanism for the transcriptional regulation of STIM1 in TGF-β signaling

We further investigated the mechanism for the TGF-β-induced downregulation of STIM1 in MDA-MB-231 cells. The expression of the *STIM1* gene was largely regulated at transcriptional and translational levels. The STIM1 mRNA expression level decreased after TGF-β (5 ng/ml) treatment for 24 and 48 h (Figure 4A); thus, we focused on the transcriptional regulation of *STIM1* following TGF-β treatment. The zinc-finger protein Wilm's tumor suppressor 1 (WT1) reportedly inhibits *STIM* 1 expression, whereas early growth response 1 (EGR1) drives *STIM1* expression in HEK293 cells [29]. We determined whether WT1 and EGR1 were involved in the TGF-β-induced reduction in the *STIM1* mRNA expression level. The relative mRNA expression levels of *WT1* and *EGR1* were quantified by qRT-PCR in TGF-β-treated samples, with untreated samples serving as controls. As shown in Figure 4D and Supplementary Figure S4J, after TGF-β treatment for 24 h, the WT1 mRNA expression level significantly increased (Figure 4D), whereas that for EGR1 did not change (Supplementary Figure S4J) compared with control levels. We performed WT1 knockdown experiments to confirm the role of this zinc-finger protein in the TGF-β-induced downregulation of STIM1. Firstly, we used three pairs of WT1 siRNAs to determine the knockdown efficiency. Transient transfection experiments showed that the siWT1-3 siRNA effectively silenced the WT1 mRNA expression level (Supplementary Figure S6). Thus, we chose the siWT1-3 siRNA to perform the following experiments. After knockdown of WT1, the TGF-β-induced downregulation of STIM1 mRNA and protein levels were lower than those of the negative control (Figure 4E-4G). These results indicated that the knockdown of WT1 expression partially restored the TGF-β-induced downregulation of STIM1 in MDA-MB-231 cells. These results suggest that the TGF-β-induced upregulation of WT1 expression might lead to the downregulation of STIM1 in MDA-MB-231 cells.

### Stable overexpression of STIM1 abolishes the TGF-β-induced suppression of cell proliferation in MDA-MB-231 cells

**Figure 5:**
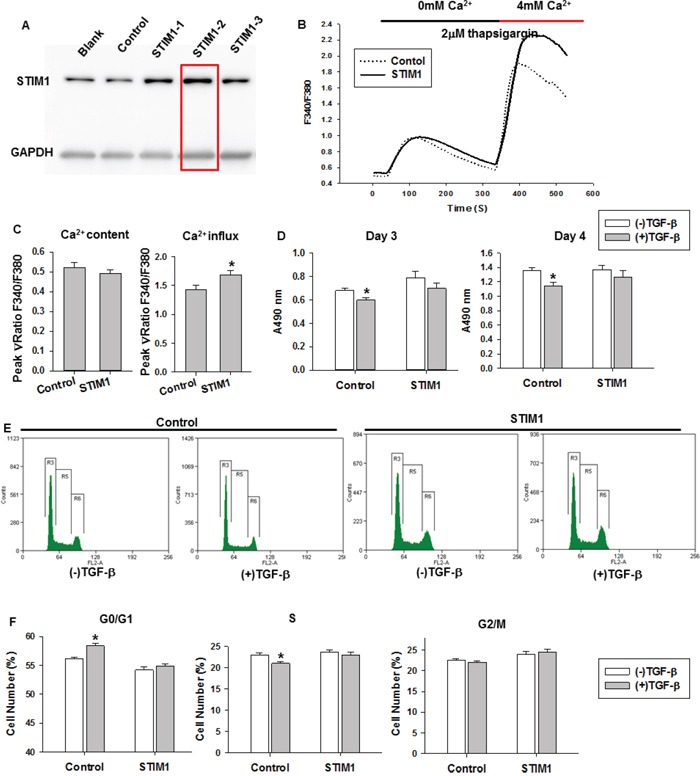
The effects of STIM1 overexpression on TGF-β-induced cell proliferation and cell cycle **A.** Western blot results showed the expression level of STIM1 in stable STIM1-overexpressing, tdTomato (control), and mock MDA-MB-231 cell lines. **B.** The average traces of intracellular Ca^2+^ signals were monitored in stable STIM1-overexpressing or control MDA-MB-231 cells by Fura 2-AM imaging (more than 30 cells each group). **C.** The results of a quantitative analysis of Ca^2+^ content and Ca^2+^ influx induced by TG and 4 mM Ca^2+^ respectively. **D.** The MTT assay results in stable STIM1-overexpressing and control cells with TGF-β treatment for 3 and 4 days. **E.** The cell cycle distribution in stable STIM1-overexpressing and control groups with TGF-β treatment for 3 days. **F.** The statistical analysis of cell cycle distribution at G0/G1, S and G2/M phases in stable STIM1-overexpressing and control cells with TGF-β treatment for 3 days. For this assay, bar graphs showed means ± SE for three independent experiments. Significance was assessed using student's t-test, *P < 0.05, **P < 0.01.

To investigate whether STIM1 was directly involved in the TGF-β-induced suppression of cell proliferation, we constructed the STIM1-IRES-tdTomato lentiviral vector and packaged the lentivirus to infect MDA-MB-231 cells. The tdTomato lentivirus was used as the control. The infection efficiency is presented in Supplementary Figure S7. To obtain single clones of STIM1-overexpressing cells, the lentivirus-infected cells were sorted using fluorescence activated cell sorting. The sorted 192 STIM1-IRES-tdTomato-positive cells were seeded in two 96-well plates and cultured for 20 days. We established 23 cell lines stably overexpressing STIM1 and used Western blot analysis to examine STIM1 overexpression in these cell lines. The results showed that the STIM1 protein level in STIM1-overexpressing cells was two times higher than that in cells from the control and mock groups (Figure 5A). Clone number 2 (STIM1-2) was selected to perform the following experiments. To investigate the function of overexpressed STIM1, SOCE was analyzed using Fura 2-AM imaging. As shown in Figure 5B, TG (2 μM) was added to the extracellular solution 30 s after incubation in a Ca^2+^-free extracellular solution, and 4 mM CaCl_2_ was added to the extracellular solution after 300 s of TG treatment. As shown in Figure 5C, the control and STIM1-overexpressing groups showed no apparent difference in TG-induced Ca^2+^ release from the endoplasmic reticulum; however, Ca^2+^ influx significantly increased in the STIM1-overexpressing group. To determine whether STIM1-overexpressing affects cell proliferation and cell viability, we measured the cell viability with an MTT assay. The absorbance values were normalized to the absorbance value at 6 h after seeding. As shown in Supplementary Figure S8A, cell proliferation and cell viability increased in STIM1-overexpressing cells. These observations confirmed that STIM1 was overexpressed in clone number 2 and that STIM1 regulated SOCE signaling.

Based on these results, we tested whether STIM1 overexpression could restore the TGF-β-induced suppression of cell proliferation. For this assay, STIM1-overexpressing and control cells were treated with TGF-β (5 ng/ml) for 3 and 4 days, and cell viability was measured with an MTT assay. As shown in Figure 5D, TGF-β significantly decreased cell proliferation in control cells, but the reduction was not significant in STIM1-overexpressing cells. We then treated STIM1-overexpressing and control cells with different concentrations of SKF96365, and cell viability was measured with an MTT assay. Although SKF96365 (1.5 μM and 2 μM) significantly decreased cell proliferation in both STIM1-overexpressing and control cells (Supplementary Figure S8B), STIM1-overexpressing cells were more sensitive to the Ca^2+^ concentration than control cells (Supplementary Figure S8C). We also performed cell cycle analysis. STIM1-overexpressing and control cells were cultured in Dulbecco's modified Eagle's medium with 10% fetal bovine serum in the presence or absence of TGF-β (5 ng/ml). After culturing for 3 days, the cell cycle distribution was analyzed using flow cytometry. TGF-β treatment significantly increased the percentage of cells at the G0/G1 phase in the control group, but this effect was not observed in STIM1-overexpressing cells (Figure 5E and 5F). Thus, STIM1 overexpression in MDA-MB-231 cells restored the TGF-β-induced suppression of cell proliferation. These results indicate that STIM1 plays an important role in the TGF-β-induced suppression of cell proliferation.

### Crosstalk between TGF-β signaling and SOCE in ERK signaling and cell cycle proteins

**Figure 6:**
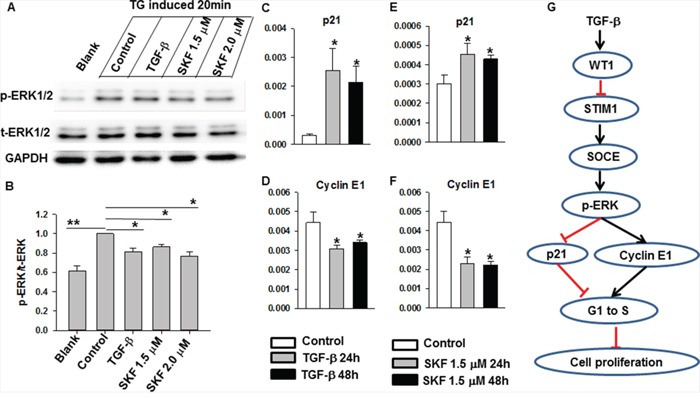
The effects of TGF-β and SKF96365 treatment in ERK and cell cycle-related signaling **A.** Western blot results showed that thapsigargin increased the phosphorylation of ERK1/2 and the treatment of TGF-β and SKF96365 decreased the phosphorylation of ERK1/2. **B.** The statistical results of p-ERK1/2 protein expression level referenced to total ERK1/2. **C-D.** TGF-β treatment for the indicated time induced the altered expression of cell cycle-related molecules p21 and cyclin E1. **E-F.** SKF96365 treatment for the indicated time induced the altered expression of cell cycle-related molecules p21 and cyclin E1. **G.** Model to depict STIM1 involving in TGF-β-induced suppression of cell proliferation in breast cancer cells. For this assay, bar graphs showed means ± SE for at least three independent experiments. Significance was assessed using one-way ANOVA with Bonferroni's multiple comparisons post-tests *P < 0.05.

The Ras/Raf/MEK/ERK signaling cascade, one of the best-studied intracellular pathways, integrates a wide variety of extracellular stimuli into key biological responses that control cell proliferation and differentiation [17, 30]. In noncanonical TGF-β signaling, TGF-β alters cell behavior through its activation of key proteins, such as the mitogen-activated protein (MAP) kinase ERK1/2, within the Smad2/3-independent signaling cascade [17]. Furthermore, calmodulin and SOCE regulate the Ras/Raf/MEK pathway and affect ERK1/2 activity [31, 32]. In a previous study, we found that AKT/PKB and ERK regulate the cell cycle through Src signaling [33]. Yang and colleagues reported that inhibition of SOCE has no effects on the AKT/PKB pathway [34]. We analyzed ERK1/2 activity in MDA-MB-231 cells using Western blot analysis. MDA-MB-231 cells were cultured in the presence or absence of TGF-β (5 ng/ml) and SKF96365 (1.5 μM or 2 μM) for 24 h, followed by treatment with TG (2 μM) for 20 min. The cells were then harvested and lysed in RIPA buffer containing phenylmethylsulfonyl fluoride. The phosphorylated ERK1/2 level increased significantly after TG treatment. TGF-β (5 ng/ml) and SKF96365 (1.5 μM or 2 μM) treatment decreased TG-induced ERK1/2 phosphorylation (Figure 6A and 6B). These results indicated that both TGF-β and SKF96365 decreased TG-induced ERK1/2 phosphorylation, which in turn suppressed cell proliferation. As shown in Figure 3C and 3D, the treatment with either TGF-β or SKF96365 induced G0/G1 arrest in MDA-MB-231 cells. To further understand the suppression of the G1 to S transition, we performed qRT-PCR to quantify the mRNA expression levels of the cell cycle proteins p21, CDK4, and cyclin E1 [35, 36]. As shown in Figure 6C and 6D, after TGF-β treatment for 24 and 48 h, the mRNA expression levels of p21 and cyclin E1 significantly increased and decreased, respectively. SKF96365 (1.5 μM) also increased the p21 mRNA expression level but decreased that for cyclin E1 (Figure 6E and 6F). By contrast, there was no change in the CDK4 mRNA expression level after TGF-β or SKF96365 treatment (data not shown). Taken together, these results indicate that a reduction in SOCE is involved in the TGF-β-induced cell cycle arrest and the suppression of cell proliferation.

## DISCUSSION

Sustaining proliferative signaling is an important biological hallmark in the multistep development of human tumors [37]. TGF-β signaling regulates a wide range of cellular behaviors, and its deregulation results in tumor development [19]. Calcium plays an important role in maintaining many physiological functions, and aberrant Ca^2+^ regulation has been linked to various diseases [1]. Our results have revealed a novel mechanism for calcium influx and TGF-β signaling in breast cancer cell proliferation.

Tumors, increasingly recognized as organs, generate a specialized microenvironment [37]. Signals from the tumor microenvironment trigger cancer progression, and crosstalk among different signaling cascades is required for this progression. Calcium signaling serves as a bridge, linking signals from the tumor microenvironment with cellular responses. The EGF-induced epithelial-mesenchymal transition is calcium-signal dependent, and the nature of that calcium signal is critically important [1, 38]. TGF-β, a multifunctional cytokine, inhibits the growth of many cancer cells, including the breast cancer cell lines MDA-MB-231 and MCF-7 [39]. In the present study, TGF-β induced cell cycle arrest at the G0/G1 phase and suppressed cell proliferation in MDA-MB-231 and MCF-7 cells. EGTA, a Ca^2+^ chelator, also induced the same effects in MDA-MB-231 cells. The removal of extracellular Ca^2+^ by EGTA impaired the TGF-β-induced arrest of the cell cycle at the G0/G1 phase and the suppression of cell proliferation in MDA-MB-231 cells (Figure 1A and 1B, 1E and 1F). These results indicate that calcium signaling plays an important role in TGF-β signaling.

Ca^2+^ influx is essential for tumor cell growth and migration [12, 40, 41]. In most cancer cells, SOCE is the major Ca^2+^ entry pathway. STIM1, an endoplasmic reticulum Ca^2+^ sensor, triggers calcium influx by activating SOCE. Recently, STIM1 has been shown to be essential for cervical cancer growth, migration, and angiogenesis as well as human glioblastoma cell proliferation and cell cycle regulation [13, 36]. Although progress has been made in defining the role of SOCE in cancer progression, the role of STIM1 and SOCE in breast cancer cell proliferation remains poorly understood, especially in the TGF-β-induced suppression of cell proliferation. In this study, we found that TGF-β treatment decreased STIM1 expression, leading to the reduction of SOCE in MDA-MB-231 cells (Figure 2A-2C, 4A-4C). The small molecule SKF96365, which specifically targets SOCE, also induced MDA-MB-231 cell cycle arrest at the G0/G1 phase and suppression of cell proliferation (Figure 3A-3D). The TGF-β-induced cell cycle arrest and suppression of cell proliferation was abolished by SKF96365. These results reveal that SOCE plays a critical role in TGF-β signaling. We also investigated the mechanism of TGF-β- and SKF96365-induced cell cycle arrest and suppression of cell proliferation. Long-term TGF-β and SKF96365 treatment decreased the Ca^2+^ influx-induced phosphorylation of ERK1/2, which increased the expression of the cell cycle-related gene p21 and decreased the expression of cyclin E1 (Figure 6A-6F). Furthermore, we established MDA-MB-231 cells stably overexpressing STIM1 and demonstrated that STIM1 overexpression restored the TGF-β-induced cell cycle arrest at the G0/G1 phase and the suppression of cell proliferation to levels near those observed in controls (Figure 5D-5F). We also followed the mechanism for the TGF-β-induced reduction in the STIM1 mRNA expression level and found that expression of the transcriptional regulator WT1 increased (Figure 4D) after TGF-β treatment, leading to the transcriptional inhibition of STIM1.

In conclusion, we have demonstrated that TGF-β induced downregulation of STIM1 expression by upregulating the transcriptional factor WT1, which resulted in reduced Ca^2+^ influx. Consistent with these findings, the effect of TGF-β on cell proliferation was nearly abolished by inhibiting Ca^2+^ influx with EGTA or SKF96365. Furthermore, STIM1 overexpression in MDA-MB-231 cells restored the TGF-β-induced cell cycle arrest and the suppression of cell proliferation to levels near those observed in controls. In this paper, we have linked TGF-β signaling with SOCE in the tumor microenvironment and elucidated the signaling mechanism that controls breast cancer cell proliferation (Figure 6G). Overall, this study reveals a new regulatory mechanism, demonstrating that SOCE and STIM1 play critical roles in TGF-β-induced cell proliferation. These findings indicate that TGF-β–STIM1 signaling may serve as a potential therapeutic target for human breast cancer.

## MATERIALS AND METHODS

### Cell lines, reagents and antibodies

All cell lines used in this work were obtained from American Type Culture Collection (Manassas, VA, USA). Cells were cultured as we previously described [42].

TGF-β was purchased from R&D systems (Minneapolis, MN, USA). SKF96365 was bought from Abcam (Abcam, UK). The calcium dye Fura 2-AM and EGTA were obtained from Invitrogen (Carlsbad, CA, USA). The antibodies against ERK1/2, p-ERK1/2 were bought from Bioworld Technology (Louis Park, MN, USA) and anti-STIM1 antibody was from Abcam (Abcam, UK). The anti-GAPDH antibody was bought from KANGCHEN BIO-TECH (Shanghai, China). HRP-conjugated goat anti-rabbit antibody was purchased from Thermo Scientific (Waltham, MA, USA).

### Immunoblotting

Immunoblotting was performed as our published protocol [42]. Briefly, cells were lysed by RIPA buffer (Beyotime Biotechnology, JiangSu, China) supplemented with PMSF (1 mM). The protein concentration was measured by the Pierce™ BCA Protein Assay Kit (Thermo Scientific). Total protein (20 μg) was separated on a 10% SDS-PAGE gel and transferred to a polyvinylidene fluoride (PVDF) membrane (Millipore, Billerica, MA, USA). Membranes were blocked by 5% skim milk in TBST, and then incubated with the primary antibodies anti-p-ERK1/2 (1:650), anti-ERK1/2 (1:650), or anti-STIM1 (1:4000) in 2% BSA solution overnight at 4°C. The membrane was incubated with HRP-conjugated secondary antibody (goat anti-rabbit antibody 1:1000) and HRP-conjugated anti-GAPDH antibody (1:10000). The immunoblotting signals were visualized with SuperSignal West Dura Extended Duration Substrate (Thermo Scientific), detected by a MiniChemi II Chemiluminescence Imaging System and analyzed using software Lane 1D (Sage Creation, Beijing, China).

### WT1 knockdown using siRNAs

The WT1 siRNAs and scrambled siRNA were purchased from Shanghai Genepharma RNAi Company (Shanghai, China). The sequences of siRNAs were shown in Supplementary Table 1. MDA-MB-231 cells were transfected with siRNA using Lipofectamine 2000 according to the manufacturer's instructions. Transfected cells were assayed on indicated time points. The qRT-PCR analysis results indicated siWT1-3 efficiently knockdown the expression of WT1. So we chose siWT1-3 to perform the following experiments. After transfection, parts of the infected cells were treated with TGF-β at 12 h and 36 h later, and the cells were harvested 60 h later, and analyzed by Western blot and qRT-PCR.

### RNA isolation and qRT-PCR

Total RNA was isolated by TRIzol reagent (Invitrogen) according to the manufacturer's introduction. Total RNA (2 μg) was used to synthesize cDNA using oligo-dT primers and SSRIII Transcriptase (Invitrogen) for reverse transcription. qRT-PCR was performed using the SYBR Green Supermix enzyme (Agilent Technologies, CA, USA) as we previously described [42]. Reactions were cycled using an Mx3000P real time PCR instrument (Agilent Technologies) with universal cycling conditions. Relative gene expression quantitation was determined with endogenous reference GAPDH gene and was analyzed using the comparative C_T_ method [43]. The quantitative PCR primers were listed in Supplementary Table 2.

### MTT cell viability assay

Cell growth curve was determined by a colorimetric MTT assay. Briefly, cells were seeded in 96-well plates (2000 cells per well). After cultured for 12 h, cells were treated with TGF-β (5 ng/ml) or EGTA (1.8 mM) and incubated for 1-4 days. Then, the cells were incubated in the medium containing a final concentration of 0.5 mg/ml of 3-(4, 5-dimethylthylthiazol-2yl-)-2, 5-diphenyl tetrazolium bromide (MTT, Sigma, Aldrich, MO, USA) solution at 37°C for 4 h. After removal of the supernatant, 150 μl DMSO (Thermo Scientific) was added to dissolve the crystals. Absorbance was measured at 490 nm using a microplate reader (Bio-Rad, Hercules, CA, USA).

### Colony formation assay

Cells were seeded in a 10 cm culture dish (200 cells per dish). Twelve hours later, cells were treated with TGF-β (5 ng/ml) or EGTA (1.8 mM). After culturing for 12 days, cell clones were washed twice with PBS, fixed with ice-cold methanol for 10 min, and stained with Giemsa for 20 min, then washed with distilled water and air-dried [36, 44]. The plates were scanned into TIFF-tag images and the colonies of area more than 20 mm^2^ were counted using the software Lane 1D in each group.

### Cell cycle analysis

The cell cycle distribution was determined by flow cytometry. Briefly, cells were seeded in 6-well plates (2 × 10^5^ cells per well). Twelve hours later, cells were treated with TGF-β (5 ng/ml), EGTA (1.8 mM), or SKF96365 (1.5 μM), respectively. The cells were harvested 72 h later, and fixed with 70% ice-cold ethanol for 30 min at 4°C, then washed with cold PBS, resuspended in 500 μl PBS containing 100 μg/ml RNase A, and incubated for 30 min at 37°C. Then the cells were incubated in 100 μl PBS containing 50 μg/ml PI for 30 min on ice in the dark, and cell cycle distribution was analyzed by a FACSCalibur flow cytometer (BD Biosciences, NJ, USA). The fractions of cells in G0/G1, S, and G2/M phases were analyzed using Summit 5.0 software.

### Lentivirus packaging and infection

The lentiviral packaging plasmids pRRE, pREV, pVsVg and pFUGW3 were provided by Dr. Chen Zhang. To construct the lentiviral plasmid expressing STIM1, the full length of STIM1 gene was amplified from the MDA-MB-231 cDNA using specific primers (Forward: 5′-ATGGATGTATGCGTCCGTCT-3′, and Reverse: 5′-CTTCTTAAGAGGCTTCTTAA-3′) and inserted into pCMV-IRES-tdTomato vector, then the full length sequence of STIM1-IRES-tdTomato or tdTomato sequence was cloned into the lentiviral plasmid vector pFUGW3. The sequences of the constructed lentiviral plasmid vectors were confirmed by sequencing. The constructed lentiviral plasmid vector (pFUGW3-STIM1-IRES-tdTomato or pFUGW3-tdTomato) and the packaging plasmids (pRRE, pREV and pVsVg) were cotransfected into HEK293T cells by Lipofectamine 2000 to produce lentiviruses. MDA-MB-231 cells were seeded in 6-well plates (2 × 10^5^ cells per well) and infected with 800 μl lentiviral supernatant containing either STIM1-IRES-tdTomato or tdTomato 12 h after seeding. The cells were infected once more 24 h later. To establish stable STIM1-overexpressing MDA-MB-231 cell colonies, the infected MDA-MB-231 cells were sorted by fluorescence activated cell sorting analysis 72 h post infection. The single cell clones were cultured for 20 days, then Western blot analysis was performed to examine the overexpression of STIM1 protein and fluorescence images were taken to examine the overexpression of tdTomato under spinning disk confocal microscope.

### Intracellular Ca^2+^ measurement

Intracellular Ca^2+^ concentration was measured by Fura 2-AM imaging with a fluorescence imaging system (Cell^R system, Olympus, Japan). In brief, cells were seeded onto coverslips and treated with TGF-β (5 ng/ml) for 24 or 48 h. For measurement of the intracellular Ca^2+^ concentration, cells were placed in a balanced salt solution (140 mM NaCl, 2.5 mM KCl, 2 mM CaCl_2_, 2 mM MgCl_2_, 12 mM D-glucose, and 10 mM HEPES, pH 7.2) [45], loaded with 1 μM Fura 2-AM in the presence of 2 mM Ca^2+^, and incubated at 37°C for 30 min. Cells were washed and the dye was allowed to de-esterify for 15 min at room temperature. Intracellular calcium measurements were performed with an excitation intensity of 340 nm and a 380 nm filter by a fluorescence imaging system using a 40× oil objective. The ratio of F340/F380 reflected the dynamic of calcium concentration. For measurement of store-operated Ca^2+^ influx, 2 μM thapsigargin was added to deplete endoplasmic reticulum calcium stores 30 S after incubation in the Ca^2+^-free extracellular solution. Ca^2+^ influx was induced by subsequent addition of 4 mM Ca^2+^ after 300 s of thapsigargin treatment. The changes in intracellular Ca^2+^ were detected as the ratio of Fura-2 fluorescence at excitation wave lengths of 340 nm and 380 nm, respectively.

### Statistical analysis

The difference between two groups was analyzed by Student's t-test, the correction more than two groups were performed by one way ANOVA, *P < 0.05 was considered statistically significant, ** represents P < 0.01.

## SUPPLEMENTARY FIGURES AND TABLES



## Abbreviations

TGF-β: transforming growth factor-β
STIM1: stromal interaction molecule 1
SOCE: store-operated calcium entry
EGF: epidermal growth factor
qRT-PCR: quantitative reverse transcription polymerase chain reaction
TRPC: transient receptor potential cation
WT1: Wilm's tumor suppressor 1
EGR1: early growth response 1
TG: thapsigargin.

## References

[1] Berridge MJ, Bootman MD, Roderick HL (2003). Calcium signalling: dynamics, homeostasis and remodelling. Nat Rev Mol Cell Biol.

[2] Vaca L (2010). SOCIC: the store-operated calcium influx complex. Cell calcium.

[3] Machaca K (2011). Ca^2+^ signaling, genes and the cell cycle. Cell calcium.

[4] Brandman O, Liou J, Park WS, Meyer T (2007). STIM2 is a feedback regulator that stabilizes basal cytosolic and endoplasmic reticulum Ca^2+^ levels. Cell.

[5] Anant BP, James WP (2005). Store-Operated Calcium Channels. Physiol Rev.

[6] Parekh AB (2010). Store-operated CRAC channels: function in health and disease. Nat Rev Drug Discov.

[7] Wang XX, Wei PL, Babu JP, Steven CS (2004). TRPC4 forms store-operated Ca^2+^ channels in mouse mesangial cells. Am J Physiol Cell Physio.

[8] Liou J, Kim ML, Heo WD, Jones JT, Myers JW, Ferrell JE, Meyer T (2005). STIM is a Ca^2+^ sensor essential for Ca^2+^-store-depletion-triggered Ca^2+^ influx. Current biology.

[9] Zhang SL, Yu Y, Roos J, Kozak JA, Deerinck TJ, Ellisman MH, Stauderman KA, Cahalan MD (2005). STIM1 is a Ca^2+^ sensor that activates CRAC channels and migrates from the Ca^2+^ store to the plasma membrane. Nature.

[10] Ritchie MF, Samakai E, Soboloff J (2012). STIM1 is required for attenuation of PMCA-mediated Ca^2+^ clearance during T-cell activation. The EMBO journal.

[11] Sebastian Schmidt GL, Guilai Liu, Wenting Yang, Sabina Honisch, Stavros Pantelakos, Christos Stournaras, Arnd Hönig, Florian Lang (2014). Enhanced Orai1 and STIM1 expression as well as store operated Ca^2+^ entry in therapy resistant ovary carcinoma cells. Oncotarget.

[12] Yang S, Zhang JJ, Huang XY (2009). Orai1 and STIM1 are critical for breast tumor cell migration and metastasis. Cancer cell.

[13] Chen YF, Chiu WT, Chen YT, Lin PY, Huang HJ, Chou CY, Chou CY, Chang HC, Tang MJ, Shen MR (2011). Calcium store sensor stromal-interaction molecule 1-dependent signaling plays an important role in cervical cancer growth, migration, and angiogenesis. Proc Natl Acad Sci.

[14] Yoshida J, Iwabuchi K, Matsui T, Ishibashi T, Masuoka T, Nishio M (2012). Knockdown of stromal interaction molecule 1 (STIM1) suppresses store-operated calcium entry, cell proliferation and tumorigenicity in human epidermoid carcinoma A431 cells. Biochemical pharmacology.

[15] Wang JY, Sun J, Huang MY, Wang YS, Hou MF, Sun Y, He H, Krishna N, Chiu SJ, Lin S, Yang S, Chang WC (2014). STIM1 overexpression promotes colorectal cancer progression, cell motility and COX-2 expression. Oncogene.

[16] Imamura T, Hikita A, Inoue Y (2012). The roles of TGF-β signaling in carcinogenesis and breast cancer metastasis. Breast cancer.

[17] Tian M, Neil JR, Schiemann WP (2011). Transforming growth factor-β and the hallmarks of cancer. Cellular signalling.

[18] Papageorgis P, Stylianopoulos T (2015). Role of TGF-β in regulation of the tumor microenvironment and drug delivery. International journal of oncology.

[19] Massague J (2008). TGF-β in Cancer. Cell.

[20] Principe DR, Doll JA, Bauer J, Jung B, Munshi HG, Bartholin L, Pasche B, Lee C, Grippo PJ (2014). TGF-β: duality of function between tumor prevention and carcinogenesis. Journal of the National Cancer Institute.

[21] Monteith GR, Davis FM, Roberts, Thomson SJ (2012). Calcium channels and pumps in cancer: changes and consequences. J Biol Chem.

[22] Capiod T (2013). The Need for Calcium Channels in Cell Proliferation. Recent Patents on Anti-Cancer Drug Discovery.

[23] Zhang J, Wei J, Kanada M, Yan L, Zhang Z, Watanabe H, Terakawa S (2013). Inhibition of store-operated Ca^2+^ entry suppresses EGF-induced migration and eliminates extravasation from vasculature in nasopharyngeal carcinoma cell. Cancer letters.

[24] Davis FM, Peters AA, Grice DM, Cabot PJ, Parat MO, Roberts-Thomson SJ, Monteith GR (2012). Non-stimulated, agonist-stimulated and store-operated Ca^2+^ influx in MDA-MB-468 breast cancer cells and the effect of EGF-induced EMT on calcium entry. PloS one.

[25] Yang HI, Tsai TY, Chiu SJ, Liu LT, Hou MF, Hsu WL, Chen BK, Chang WC (2013). Involvement of STIM1 and Orai1 in EGF-mediated cell growth in retinal pigment epithelial cells. Journal of Biomedical Science.

[26] Prevarskaya N, Skryma R, Shuba Y (2011). Calcium in tumour metastasis: new roles for known actors. Nature reviews Cancer.

[27] Minke B (2006). TRP channels and Ca^2+^ signaling. Cell calcium.

[28] Motiani RK, Zhang X, Harmon KE, Keller RS, Matrougui K, Bennett JA, Trebak M (2013). Orai3 is an estrogen receptor alpha-regulated Ca^2+^ channel that promotes tumorigenesis. FASEB J.

[29] Ritchie MF, Yue C, Zhou Y, Houghton PJ, Soboloff J (2010). Wilms tumor suppressor 1 (WT1) and early growth response 1 (EGR1) are regulators of STIM1 expression. J Biol Chem.

[30] Chambard JC, Lefloch R, Pouyssegur J, Lenormand P (2007). ERK implication in cell cycle regulation. Biochimica et biophysica acta.

[31] Neus A, Nati R, Priam V (2002). Modulation of the Ras/Raf/MEK/ERK pathway by Ca^2+^, and Calmodulin. Cellular signalling.

[32] Masanari U, Stefan F, Mariana SD, Lorenzo, Xie LH, Feng XF, Iwatsub K (2014). Store-Operated Ca^2+^ Entry (SOCE) Regulates Melanoma Proliferation and Cell Migration. PloS one.

[33] Liu X, Du L, Feng R (2013). c-Src regulates cell cycle proteins expression through protein kinase B/glycogen synthase kinase 3 beta and extracellular signal-regulated kinases 1/2 pathways in MCF-7 cells. Acta biochimica et biophysica Sinica.

[34] Sun J, Lu F, He H, Shen J, Messina J, Mathew R, Wang D, Sarnaik AA, Chang WC, Kim M, Cheng H, Yang S (2014). STIM1- and Orai1-mediated Ca^2+^ oscillation orchestrates invadopodium formation and melanoma invasion. The Journal of cell biology.

[35] Vamsidhara CD, Welshons WV, Chada SR (2006). Cell cycle proteins in normal and chemically induced abnormal secondary palate development. Human and Experimental Toxicology.

[36] Li GL, Zhang ZZ, Wang RZ, Ma WB, Yang Y, Wei JJ, Wei PY (2013). Suppression of STIM1 inhibits human glioblastoma cell proliferation and induces G0/G1 phase arrest. Journal of Experimental and Clinical Cancer Research.

[37] Hanahan D, Weinberg RA (2011). Hallmarks of cancer: the next generation. Cell.

[38] Davis FM, Azimi I, Faville RA, Peters AA, Jalink K, Putney JW, Goodhill GJ, Thompson EW, Roberts SJ, Monteith GR (2014). Induction of epithelial-mesenchymal transition (EMT) in breast cancer cells is calcium signal dependent. Oncogene.

[39] Brown KA, Aakre ME, Gorska AE, Price JO, Eltom SE, Pietenpol JA, Moses HL (2004). Induction by transforming growth factor-β1 of epithelial to mesenchymal transition is a rare event in vitro. Breast cancer research.

[40] Kim JH, Lkhagvadorj S, Lee MR, Hwang KH, Chung HC, Jung JH, Cha SK, Eom M (2014). Orai1 and STIM1 are critical for cell migration and proliferation of clear cell renal cell carcinoma. Biochem Biophys Res Commun.

[41] Hou MF, Kuo HC, Li JH, Wang YS, Chang CC, Chen KC, Chen WC, Chiu CC, Yang S, Chang WC (2011). Orai1/CRACM1 overexpression suppresses cell proliferation via attenuation of the store-operated calcium influx-mediated signalling pathway in A549 lung cancer cells. Biochimica et biophysica acta.

[42] Liu X, Feng R, Du L (2010). The role of enoyl-CoA hydratase short chain 1 and peroxiredoxin 3 in PP2-induced apoptosis in human breast cancer MCF-7 cells. FEBS letters.

[43] Livak KJ, Schmittgen TD (2001). Analysis of relative gene expression data using real-time quantitative PCR and the 2^−DDCT^ Method. Methods.

[44] Ye YW, Wu JH, Wang CM, Zhou Y, Du CY, Zheng BQ, Cao X, Zhou XY, Sun M, Shi YQ (2011). Sox17 regulates proliferation and cell cycle during gastric cancer progression. Cancer letters.

[45] Jia X, Li X, Xu Y, Xu Y, Zhang S, Mou W, Liu Y, Liu Y, Lv D, Liu CH, Tan X, Xiang R, Li N (2011). SOX2 promotes tumorigenesis and increases the anti-apoptotic property of human prostate cancer cell. Journal of molecular cell biology.

