# Supplementary material for: STIM1 plays an important role in TGF-β-induced suppression of breast cancer cell proliferation
Source: Oncotarget. 2016 Feb 23;7(13):16866–78. doi: 10.18632/oncotarget.7619 (PMC4941356; doi:10.18632/oncotarget.7619)
Supplement: Supplementary file 1 [file oncotarget-07-16866-s001.pdf]

SUPPLEMENTARY FIGURES AND TABLES

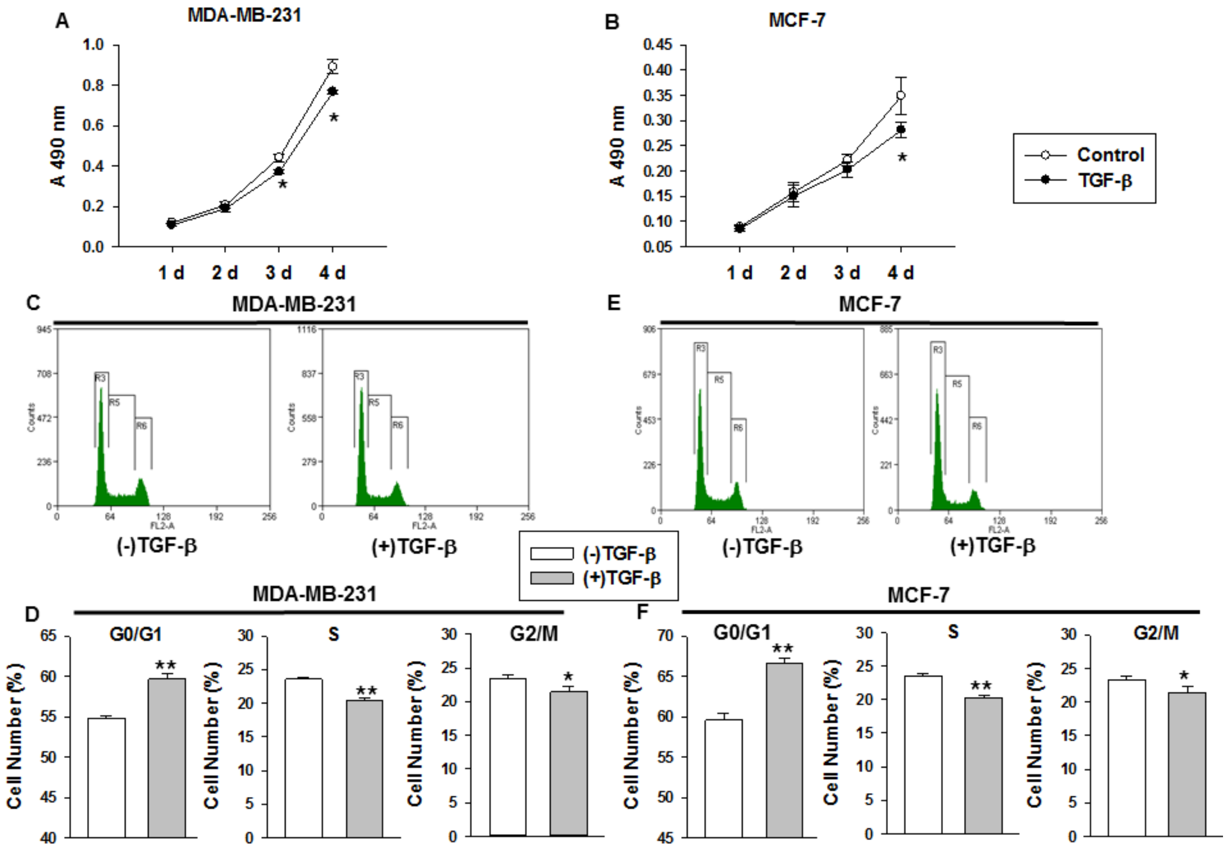

Supplementary Figure S1: TGF-β induced cell cycle arrest and suppression of MDA-MB-231 and MCF-7 cell proliferation. A-B. MDA-MB-231 and MCF-7 cell proliferation were determined by MTT assay after TGF-β (5 ng/ml) treatment for the indicated time. **C.** and **E.** Cell cycle distribution was detected by flow cytometry in MDA-MB-231 and MCF-7 cells after TGF-β (5 ng/ml) treatment for 3 days. **D.** and **F.** The statistical analysis of MDA-MB-231 and MCF-7 cell cycle distribution at G0/G1, S and G2/M phases in TGF-β (5 ng/ml) treatment and control groups. For this assay, bar graphs showed means ± SE for at least three independent experiments. Significance was assessed using student's t-test *P < 0.05, **P < 0.01 compared with control group.

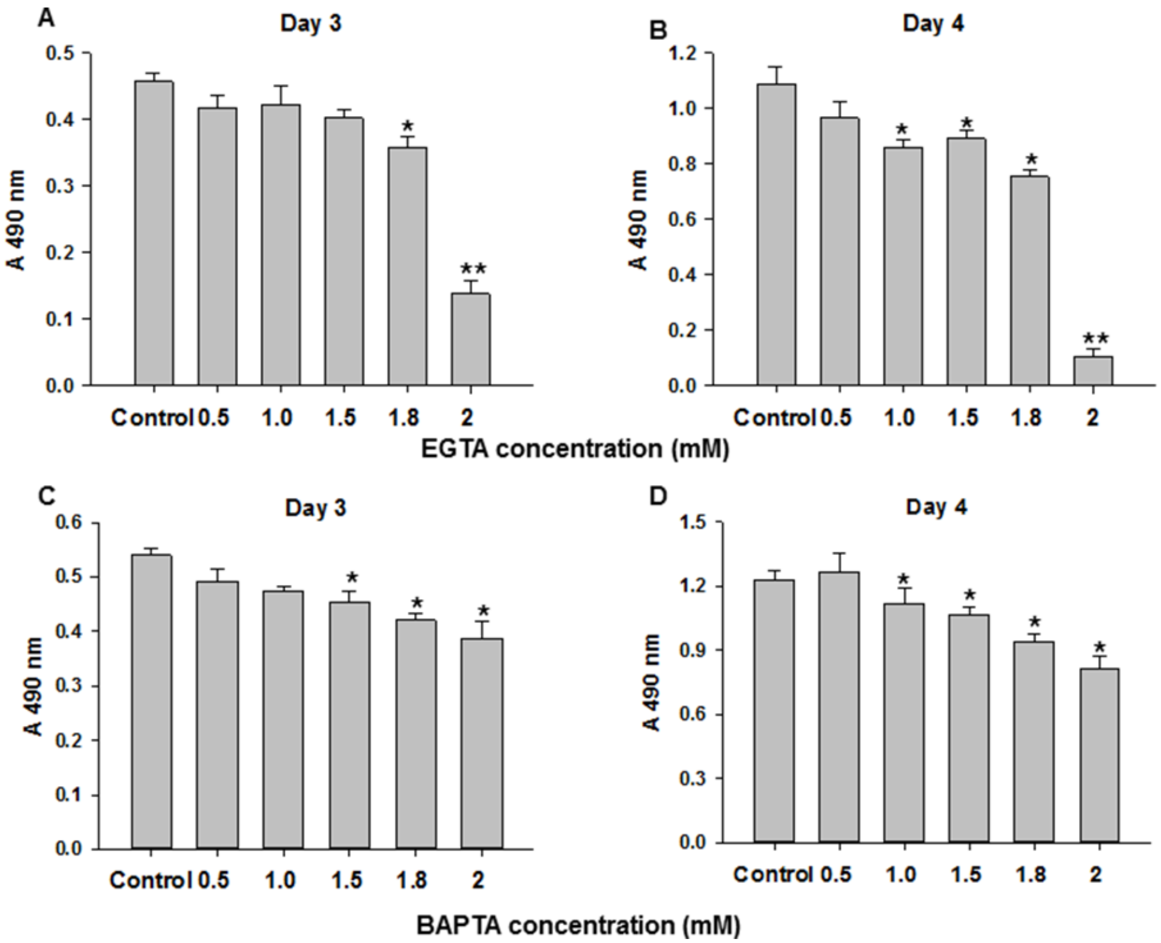

Supplementary Figure S2: The MDA-MB-231 cell proliferation assay in various concentrations of EGTA or BAPTA treatment. A-B. MDA-MB-231 cells were treated with various concentrations of EGTA for 3 and 4 days. **C-D.** MDA-MB-231 cells were treated with various concentrations of BAPTA for 3 and 4 days. The cell proliferation was determined by MTT assay. For this assay, bar graphs showed means ± SE for at least three independent experiments. Significance was assessed using one-way ANOVA with Bonferroni's multiple comparisons post-tests *$P < 0.05$.

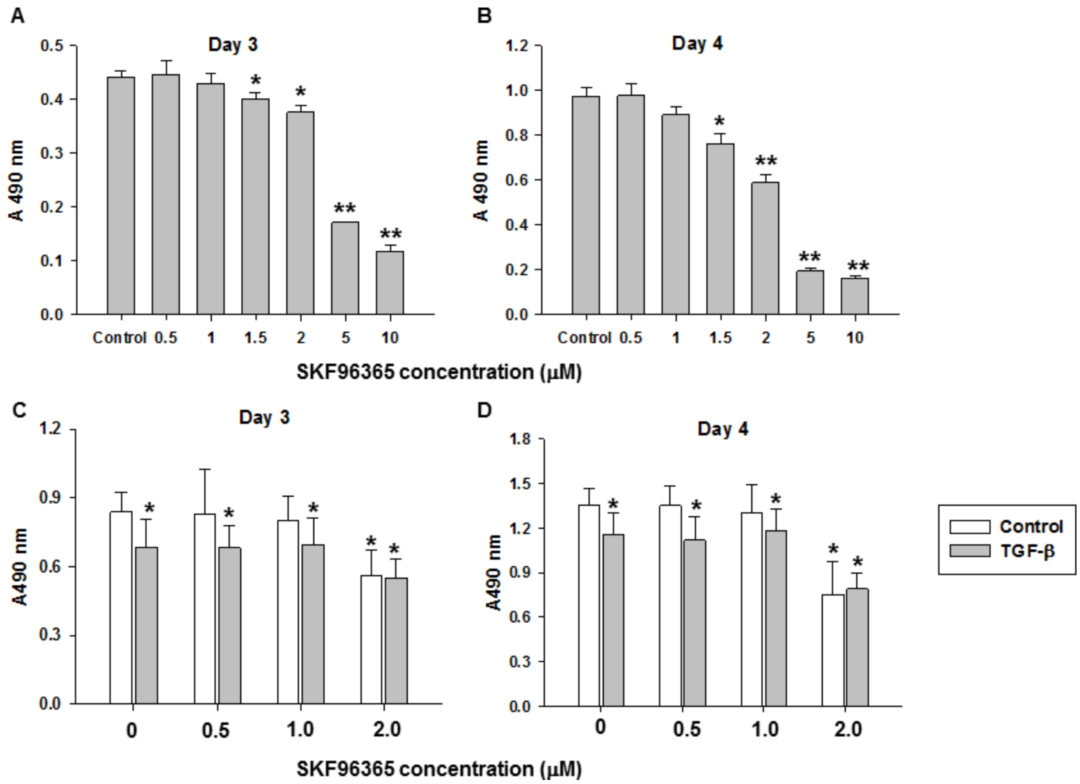

Supplementary Figure S3: The MDA-MB-231 cell proliferation assay in TGF-β and various concentrations of SKF96365 treatment. A-B. MDA-MB-231 cells were treated with various concentrations of SKF96365 for 3 and 4 days, respectively. **C-D.** MDA-MB-231 cells were treated with various concentrations of SKF96365 with or without TGF-β, respectively, for 3 and 4 days. *P<0.05 VS. Control and SKF 0 μM. The cell proliferation was determined by MTT assay. For this assay, bar graphs showed means ± SE for at least three independent experiments. Significance was assessed using one-way ANOVA with Bonferroni's multiple comparisons post-tests.

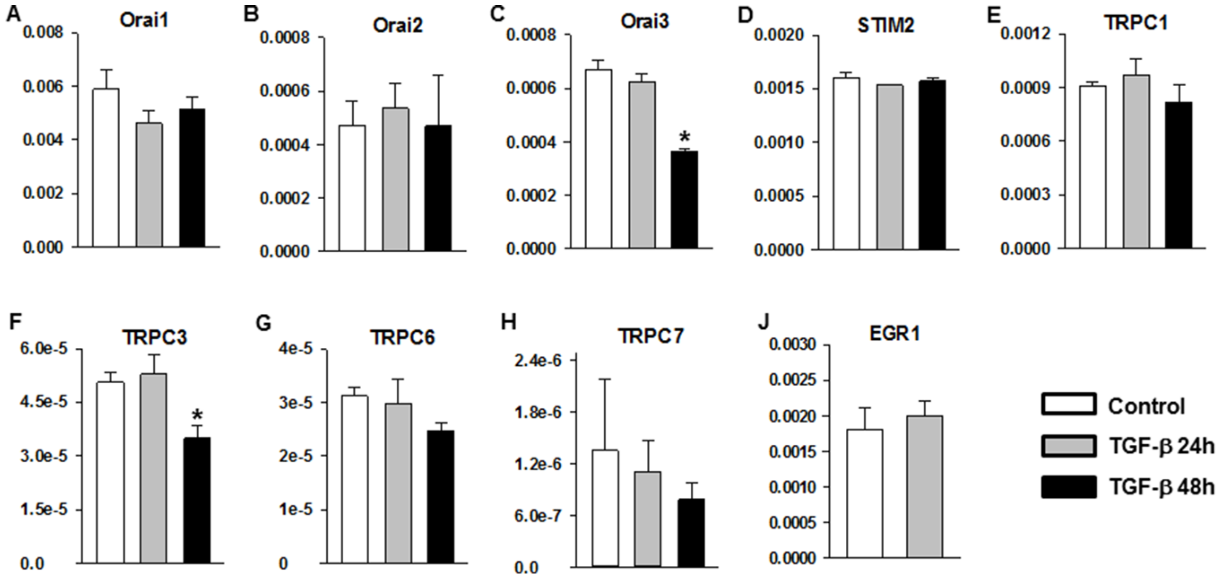

Supplementary Figure S4: The role of TGF-β on some SOC-related gene-and *EGR1* gene expression in MDA-MB-231 cells. **A.** *Orai1*. **B.** *Orai2*. **C.** *Orai3*. **D.** *STIM2*. **E.** *TRPC1*. **F.** *TRPC3*. **G.** *TRPC6*. **H.** *TRPC7*. **J.** *EGR1*.

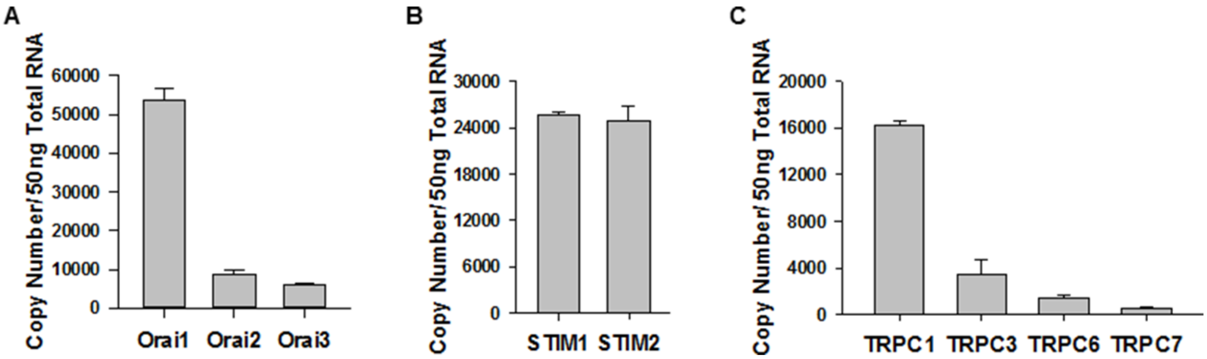

Supplementary Figure S5: The copy numbers of SOC-related genes in MDA-MB-231 cells were analyzed by absolute quantitative RT-PCR. A. The copy number of *Orai* channels in 50 ng total RNAs. **B.** The copy number of *STIM* sensors in 50 ng total RNAs. **C.** The copy number of *TRPC* channels in 50 ng total RNAs.

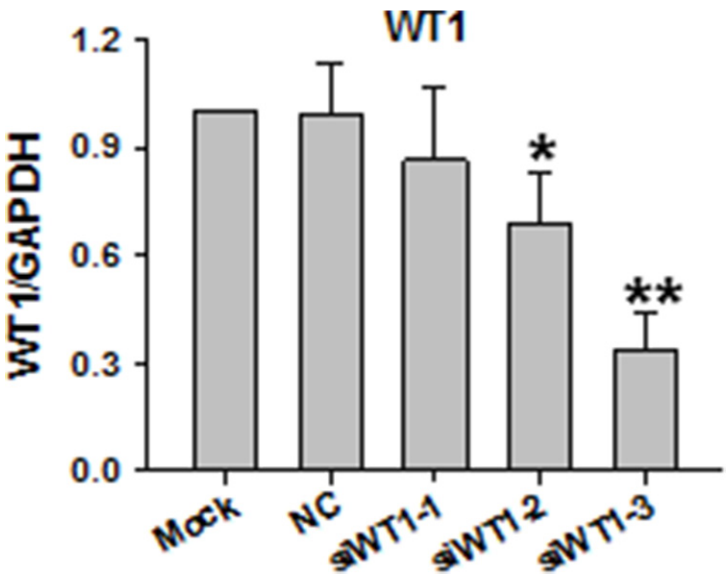

Supplementary Figure S6: The knockdown efficiency of WT1 siRNAs. The qRT-PCR results showed that transient transfection with WT1 siRNA decreased the WT1 expression in mRNA level.

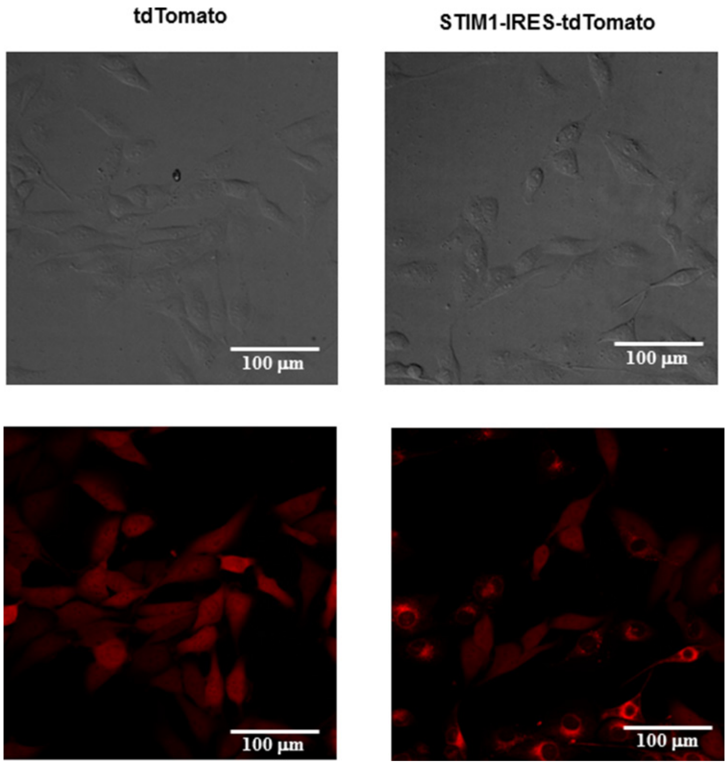

Supplementary Figure S7: The fluorescence image of lentivirus infected MDA-MB-231 cells. The fluorescence images were taken after lentiviral infection for 72 h under a spinning disk confocal microscope.

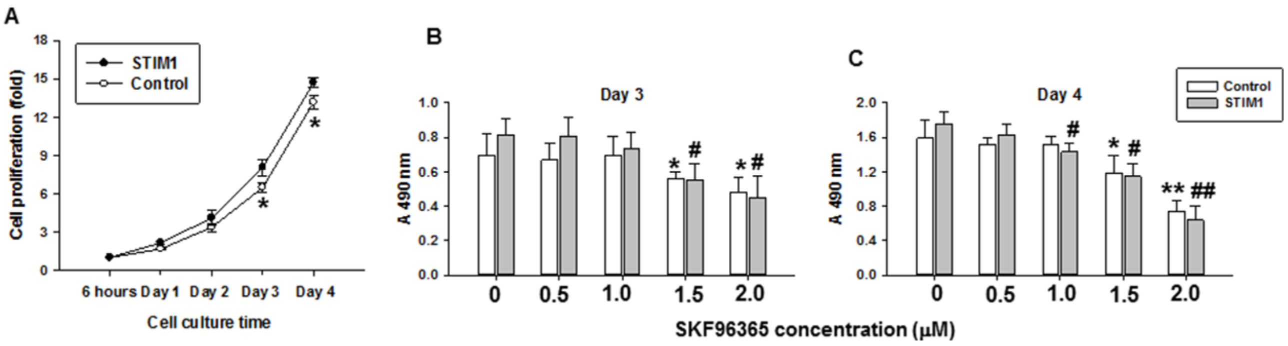

Supplementary Figure S8: The cell proliferation assay of STIM1-overexpressing and control cells. **A**. The results of proliferation assay. After culturing for 6 h, 1 day, 2 days, 3 days, 4 days, cell viability was detected using MTT assay. **B-C**. The results of MTT assay. STIM1-overexpressing and control cells were treated with various concentrations of SKF96365 for 3 and 4 days, respectively. *P<0.05 and ** P<0.01 VS. Control and SKF 0 µM, #P<0.05 and ## P<0.01 VS. STIM1-OE and SKF 0 µM. The cell proliferation was determined by MTT assay. For this assay, bar graphs showed means ± SE for at least three independent experiments. Significance was assessed using one-way ANOVA with Bonferroni's multiple comparisons post-tests.

Supplementary Table S1: siRNAs sequencs used in this paper

	siRNA sequence (5'----3')
Negative Control	Sense 5'-UUC UCC GAA CGU GUC ACG UTT-3'
	Antisense 5'-ACG UGA CAC GUU CGG AGA ATT-3'
siWT1-1	Sense 5'-CCU ACA GCA GUG ACA AUU UTT-3'
	Antisense 5'-AAA UUG UCA CUG CUG UAG GTT-3'
siWT1-2	Sense 5'-GCU UAC CCA GGC UGC AAU ATT-3'
	Antisense 5'-UAU UGC AGC CUG GGU AAG CTT-3'
siWT1-3	Sense 5'-GGA GAC AUA CAG GUG UGA ATT-3'
	Antisense 5'-UUC ACA CCU GUA UGU CUC CTT-3'

Supplementary Table S2: qRT-PCR primers used in this paper

Gene name	Primer sequence (5'----3')	Product length	Accession NO.
Orai1	F: ATGGTGGCAATGGTGGAG	115bp	NM_032790
	R: CTGATCATGAGCGCAAACAG		
Orai2	F: CCGTGCTTGGCATCCTACTCTT	184bp	NM_001126340.2
	R: GTGAAGACCACGAAGATGAGGC		
Orai3	F: CCTTGCTGAAGTTGTCCTGGTT	161bp	NM_152288.2
	R: ACGCAGAGGACCGTGGGAGATT		
STIM1	F: TGTGGAGCTGCCTCAGTATG	112bp	NM_003156
	R: CTTCAGCACAGTCCCTGTCA		
STIM2	F: GGATTCGCCTGTAACTGTGGAT	151bp	NM_001169118.1
	R: GATGCCACTGGAAAGCTGGTTC		
TRPC1	F: GTCGTGGTTGTGATTGTGCTTAC	219bp	NM_001251845.1
	R: GCAAATCCACTTACTGAGGCTAC		
TRPC3	F: CTGCCGAGACTCAGAAGAGGTAG	115bp	NM_003305.2
	R: CTTAATGGCAAGTTTGACACGA		
TRPC4	F: CACAATACAATCTGCGAATGCC	144bp	NM_016179.2
	R: TGATCTCGGATGAATCAGGGTG		
TRPC5	F: TGTGGGATGGTGGATTTACTGA	225bp	NM_012471.2
	R: ACAGGGATATGAGACGCAACGA		
TRPC6	F: TGGGCACAATAAACAACCAAGTA	215bp	NM_004621.5
	R: CTTCAAGGAGTTCATAGCGGAGAC		
TRPC7	F: AACCTGCTAGATTTCGGGATGCT	138bp	NM_020389.2
	R: AAGCGAGACATTGTGCAGCGTGT		
p21	F: ACCTGTCACTGTCTTGTACCCTTG	117bp	NM_000389.4
	R: AGAAATCTGTCATGCTGGTCTGC		
CyclinE1	F: TTCAGGGTATCAGTGGTGCGACAT	183bp	NM_001238.2
	R: TTCTTTGCTCGGGCTTTGTCCAG		
WT1	F: GAAATGGACAGAAGGGCAGAGC	173bp	NM_001198551.1
	R: CAGATGCCGACCGTACAAGAGT		
EGR1	F: TGCCCAGTGGAGTCCTGTGAT	208bp	NM_001238.2
	R: CGCTCCTGGCAAACTTTCTTC		
GAPDH	F: TGAAGGTCGGAGTCAACGGAT	225bp	NM_002046.4
	R: CCTGGAAGATGGTGATGGGAT		
